# Genetic mapping reveals QTLs associated with HLB response determined by *Candidatus* Liberibacter asiaticus quantification in a *Citrus sunki* × *Poncirus trifoliata* segregating population

**DOI:** 10.3389/fpls.2026.1864793

**Published:** 2026-08-17

**Authors:** Luciane Santini-Gazaffi, Rodrigo Gazaffi, Sinara Oliveira de Aquino, Dário Grattapaglia, Marinês Bastianel, Mariângela Cristofani-Yaly, Marcos Antonio Machado

**Affiliations:** 1 Instituto Agronômico de Campinas, Centro APTA Citros Sylvio Moreira, Sao Paulo, Brazil; 2 Universidade Federal de São Carlos, Centro de Ciências Agrárias, Departamento de Biotecnologia e Produção Vegetal e Animal, Sao Paulo, Brazil; 3 Empresa Brasileira de Pesquisa Agropecuária (EMBRAPA) - Recursos Genéticos e Biotecnologia, Brasília, Brazil

**Keywords:** citrandarins, greening, high-throughput markers, phenotyping, QTL mapping, quantitative PCR (qPCR), SNPs

## Abstract

**Introduction:**

Huanglongbing (HLB) is a devastating disease impacting citrus orchards worldwide. Identifying candidate genes and/or genomic regions that significantly influence plants' response to HLB is essential for breeding programs. Nevertheless, this remains a challenge, especially during the early stages of the citrus-pathogen interaction, when phenotypically distinguishing tolerant genotypes is not trivial. This work focused on identifying genomic regions and candidate genes associated with citrus response to *Candidatus* Liberibacter asiaticus (*C*Las).

**Methods:**

Quantitative trait loci (QTL) mapping was performed in a citrandarin population (278 individuals) derived from a fullsib cross between susceptible *Citrus sunki* (CS) and tolerant *Poncirus trifoliata* (PT). The mapping population was genotyped using DArTseq markers and the SNPs obtained from them (DArTseq-derived SNPs), as well as the EMBRAPA Multispecies 65K Illumina Infinium® chip. Phenotyping was based on four individual trials (two in a greenhouse and two in field conditions). QTL mapping was based on composite interval mapping implemented in the fullsibQTL software, with the cycle threshold (Ct) of quantitative PCR (qPCR) as the trait of interest for assessing *C*Las quantification in infected tissues, which can be evaluated in plants at any developmental stage. The biological function of QTLs was investigated by gene annotation using the chromosome-level phased *Citrus sinensis* genome (assembly DVS_A1.0) as the reference. The candidate genes were functionally annotated using Blast2Go software.

**Results:**

A predominance of markers in a 1:1 ratio was observed, resulting in two maps, one for each parent. The linkage map had 5464 cM (1872 markers) for CS and 4794 cM (1340 markers) for PT. A total of 44 QTLs were mapped: 25 for the CS map (13 for field and 12 for greenhouse conditions), of which 17 were located at gene sites; and 19 were mapped for the PT (nine for field and 10 for greenhouse conditions), of which 16 were located at gene sites.

**Discussion:**

Several genes associated with the identified QTLs are involved in stress responses, making them potential targets for further functional validation. These genomic regions and candidate genes provide valuable targets for future incorporation into citrus breeding programs aimed at developing HLB tolerance.

## 1 Introduction

Huanglongbing (HLB) is a severe disease caused by the bacteria *Candidatus Liberibacter* spp., transmitted by the psyllid *Diaphorina citri* (Hall et al., 2013). First reported in Brazil in 2004 (Coletta-Filho et al., 2004), HLB remains a significant threat to global citrus production (Lovelace et al., 2025).

The search for resistant varieties that meet producer demand has thus become a top priority in citrus cultivation. For this, some aspects may be considered, i.e., the psyllid interaction with host plants and/or the dynamics of *C*Las in host plants. Considering the plant-pathogen interaction, the severity of disease symptoms in commercial citrus species has been extensive documented (Boscariol-Camargo et al., 2010; Albrecht and Bowman, 2012; Boava et al., 2017; Nehela and Killiny, 2020), such as physiological, cellular, and molecular disorders, such as phloem collapse, root system disintegration, and drastic disruption in carbohydrate translocation, consequently generating small and deformed fruits, premature fruit drop, aborted seeds, and bitter juice; resulting in the agroeconomic decline of citrus crop (Li et al., 2020).

Fully commercial-resistant genotypes to HLB have not been reported, to our knowledge, which remains an issue. However, several studies have demonstrated tolerance in citrus-relative genera, including *Poncirus* (Ramadugu et al., 2016), especially *Poncirus trifoliata*. Studies have shown that the trifoliate orange group exhibits higher tolerance to HLB than other citrus groups (Folimonova et al., 2009; Boscariol-Camargo et al., 2010; Albrecht and Bowman, 2012; Boava et al., 2017; Huang et al., 2018, 2023). This species does not exhibit any symptoms or significant starch accumulation during bacterial growth; starch accumulation remains low or almost absent (Boava et al., 2017). Citrandarins, hybrids resulting from controlled crosses between *C. sunki* (a susceptible phenotype) and *P. trifoliata*, show high variability in *C*Las quantification by qPCR Ct (cycle threshold) and starch content. According to Boava et al. (2017), the susceptible group tested positive for HLB and displayed significant starch accumulation, while the tolerant group was also HLB-positive but showed no significant difference in starch levels. The resistant group, however, tested negative for HLB and presented no changes in starch content. Nonetheless, considerable work still needs to be done before a citrus-resistant variety release.

One of the challenges for citrus breeding programs is phenotyping for greening response and correctly identifying resistant and/or tolerant genotypes, given the wide phenotypic variation observed in the germplasm (Yu et al., 2022). Several characteristics, such as visual scores of infected plants (Yu et al., 2022), *C*Las quantification determined by qPCR (Stover and McCollum, 2011; McCollum et al., 2014), starch and callose accumulation in phloem (Chen et al., 2022b; Yao et al., 2024), variations in morphological traits, photosynthetic performance (Chen et al., 2022b; Yu et al., 2022), yield, and quality of production (Yu et al., 2022) can be used to screen infected plants. Nevertheless, most of these traits manifest in the late stages of the citrus-*C*Las interaction in adult plants, as evidenced by visual symptoms and decreased yield and fruit quality. Considering that early selection of promising genotypes is desirable in breeding programs, screening based on bacterial multiplication in plant tissues, using Ct values or *C*Las titer estimated by qPCR, is an advantageous trait that allows early selection, even in the citrus juvenile stage.

Given the complexity of HLB response phenotyping, identifying molecular markers associated with tolerance is highly desirable and may improve germplasm screening and accelerate breeding programs. However, breeding programs aimed at developing new citrus varieties should also consider a range of agronomic traits, including resistance to HLB. Since HLB is a quantitative trait influenced by multiple genetic factors, further research is needed to understand its genetic architecture, as investigated by Diaz et al. (2025). Citrus responses to stress induced by pathogens, like *C*Las or environmental factors, alter the expression of numerous genes in a complex and dynamic manner, aiming to mitigate the detrimental effects they encounter. Studies focusing on transcriptomics have yielded insights into the modulation of thousands of genes as a response to HLB in citrus (Liu et al., 2024a; Alves et al., 2025). However, identifying candidate genes that significantly influence the defense response and can be incorporated into biotechnology and breeding programs to increase tolerance remains a challenge. In this context, Quantitative Trait Loci (QTL) mapping provides valuable support for decision-making by identifying regions associated with this trait.

The comprehension achieved through QTL mapping (Curtolo et al., 2020; Huang et al., 2023), promises to be applied in practice by breeders who pyramid alleles of interest into new varieties (Ezra and Carmi, 2025) as well as in assisted selection in breeding programs. These genomic regions can be revealed using molecular markers under different technologies (Tripodi, 2023). Previously, these QTLs were identified using anonymous markers based on DNA fragments. However, advances in sequencing technologies and functional genomics have enabled in-depth investigation of candidate genes associated with QTLs (Tyagi et al., 2024). The concept of QTL mapping is the association between phenotypes and genomic regions to identify regions associated with an interesting trait, and several methods have been developed across different segregating populations (Doerge, 2002). Usually, for non-inbred populations, QTL mapping is based on biparental crosses (Gazaffi et al., 2014), where, over time, results have been reported for several traits in citrus (Huang et al., 2018; Curtolo et al., 2020; Khefifi et al., 2022; Matsumoto et al., 2022; Huang et al., 2023; Kubo et al., 2023). Also, when expression studies are combined with QTL mapping, the concept of expression quantitative trait loci (eQTL) emerges. eQTLs are gene regions identified by the abundance of specific gene transcripts. Integrating genotypic and quantitative transcription data analysis can facilitate the identification of genes associated with specific phenotypes (Soratto et al., 2020). All this information is useful, as HLB can reveal a complex network involved in its gene expression (Yu et al., 2022), which can also be influenced by environmental conditions. Understanding the components involved in this interaction could ultimately lead to the development of genotypes that are either resistant or highly tolerant to the *C*Las.

In this scenario, we present an update of the genetic map for a fullsib population of *Citrus sunki* × *Poncirus trifoliata* (Curtolo et al., 2017), a citrandarin population, by merging genotypic datasets from multiple high-throughput platforms. We also performed QTL mapping using four assays for HLB response, based on *C*Las quantification determined by Ct values of quantitative PCR (qPCR).

## 2 Materials and methods

### 2.1 Plant material and genotyping

The mapping population was comprised of 278 individuals obtained from a full-sib cross (also named as F1 population) between the female parent *Citrus sunki* Hort. ex Tan. and the male parent *Poncirus trifoliata* (L.) Raf. cv. Rubidoux were genotyped, used in mapping analyses, and evaluated for their response to *C*Las infection. Healthy leaf tissue was collected for genotyping, and leaf tissue from infected clones was used in quantitative PCR analyses to evaluate the plants’ response to the bacteria. To prevent bias in *C*Las quantification caused by non-uniform bacterial distribution in plant tissues, two leaves were sampled from similar positions on all four sides of each plant and combined for DNA extraction, as described in detail by Soratto et al. (2020).

The genotyping data were based on three high-throughput platforms. Dominant markers were obtained from 27, 960 loci sampled by DArTseq markers, and codominant markers were generated from 17, 482 SNPs obtained from DArTseq and 3, 608 citrus SNPs from the EMBRAPA multispecies 65K Illumina Infinium^®^ chip (Grattapaglia et al., 2017; Lopes et al., 2022). The markers were coded according to the polymorphisms identified in the parents and their segregation in the population, following the notation proposed by Wu et al. (2002a), briefly described below. DArT-seq identified all possible segregations for dominant markers in the F1 population, which were 1:1 ratio in the marker type D1.13 (polymorphism only in the female parent, i.e., ao × oo) and the marker type D2.18 (polymorphism only in the male parent, i.e., oo × ao) and the segregation 3:1 in the C.8 markers (both parents present the polymorphism, i.e., ao × ao). Considering the SNP markers, the 1:1 and 1:2:1 segregation ratios were verified, which were D1.10 (polymorphism in *C. sunki*, i.e., ab × aa), D2.15 (polymorphism in *P. trifoliata*, i.e., aa × ab), and B3.7 (polymorphism in both parents, i.e., ab × ab).

### 2.2 Genetic mapping

First, BLASTn was used to compare the nucleotide sequence of all genetic markers with the *Citrus sinensis* reference genome, version DVS_A1.0, available at NCBI. Markers with a single hit in the *C. sinensis* genome had their physical position included in the raw data for genetic mapping. The genomic position of markers not identified by BLASTn was considered not available (NA).

To obtain the linkage groups, the OneMap package, v.3.0.0 (Margarido et al., 2007; Taniguti et al., 2022), implemented in R, version 4.3.3 (R Core Team, 2024), was used. Due to the predominance of informative loci segregating in only one parent, the mapping strategy was similar to the pseudo-testcross (Grattapaglia and Sederoff, 1994), i.e., two separate maps were obtained (one for each parent) using markers with a 1:1 segregation ratio. Markers with 3:1 and 1:2:1 segregations, which access information from both parents, were included in both maps, allowing the identification of the corresponding linkage groups. The mapping pipeline can be detailed as follows: first, genotyping data from each sequencing platform were independently loaded into OneMap and combined into a single dataset using the “combine_onemap” function. Then, loci with more than 10% missing data were not included in the mapping procedure. The overlapping of loci obtained by the different platforms was prevented by removing redundant markers. Marker redundancy was verified using the “find_bins” function with the exact = FALSE argument, which considers the marker with the least missing data as representative of a bin. Afterward, a set of non-redundant markers was obtained with the “create_data_bins” function and used in subsequent steps. Recombination frequency was calculated using the two-point test, assuming a maximum recombination frequency of 0.3 and a LOD score of 10. Recombination frequencies were converted into map distance considering the Kosambi mapping function (Kosambi, 1943).

Markers with segregation distortion were identified by the “test_segregation” function and suppressed from mapping analysis. The linkage groups were constructed using the “group_seq” command, considering all markers allocated to each chromosome, defined by the parameter seqs = “CHROM”, and the recombination frequencies between markers. Markers without chromosomal information were added to the most likely group using the unlink.mks and repeated = FALSE criteria.

Two methods for defining the linkage-group ordering were used: one based on the physical positions of markers in the reference genome, and the other on recombination frequencies. In both cases, the initial ordering was performed considering only markers with known chromosomal positions. The first method ordered the markers by their physical positions using the commands “make_seq” and “map_avoid_unlinked”. In the second strategy, the initial ordering of the markers was obtained using the function “mds_onemap” based on the MDS method (Preedy and Hackett, 2016). Both orderings were performed using parallelization parameters (size = 20, phase_cores = 5, overlap = 10).

The linkage groups obtained in each ordering strategy were compared, and those with the shortest length in cM and the highest log(likelihood) value were chosen. If the best ordering was obtained from the genomic position, the linkage group was subjected to the marker position refinement using the “order_seq” command.

After defining the framework of each linkage group, the markers that did not have a previously defined chromosomal position were allocated to the most likely location using the “try_seq_by_seq” function. The parameters for inclusion of a marker in a group were extension up to 10 cM in length and a maximum penalty of -100 in the log(likelihood) value.

Finally, the ordering of each linkage group was verified graphically using the “rf_graph_table” command. The linkage groups with gaps greater than 30 cM were split into more saturated subgroups, thereby improving QTL mapping. Genetic maps were drawn using the LinkageMapView package (Ouellette et al., 2018) in the R platform (R Core Team, 2024).

### 2.3 Phenotyping, statistical analyses, and QTL mapping

Differences in *C*Las multiplication in hybrids from the CS × PT population were evaluated in four experiments, conducted in the experimental area at Centro de Citricultura Sylvio Moreira of the Instituto Agronômico (IAC), Cordeirópolis/SP (22°27’41.1”S 47°23’57.6” W). Two trials were implemented in a greenhouse in 2009 and 2021, respectively. Other trials were carried out under field conditions in 2014 and 2018.

In both greenhouse experiments, the *C*Las inoculation was conducted according to the methods described by Curtolo et al. (2020). Specifically, the hybrids from the study population were propagated through bud grafting onto ‘Cravo’ lemon (*Citrus limonia* Osbeck). After a period of six months, each hybrid was inoculated with two buds taken from HLB-infected ‘Pera’ sweet orange (*Citrus sinensis* (L.) Osbeck) trees, which were grafted on opposite sides of the main stem. Plants evaluated under field conditions were naturally infected with *C*Las transmitted by psyllids. Plants infection were confirmed using quantitative PCR (qPCR).

The greenhouse experiments were conducted in a completely randomized design with a maximum of four replicates. Ninety and 71 genotypes were evaluated in 2009 and 2021, respectively.

The experiments implemented under field conditions were also completely randomized, with three replicates and 66 and 265 genotypes evaluated in 2014 and 2018, respectively.

The multiplication of *C*Las in infected plant tissues was measured by quantitative PCR (qPCR). As previously mentioned, two leaves were collected from similar positions on all four sides of each plant and combined for DNA extraction and qPCR analysis, described in details by Soratto et al. (2020). Briefly, multiplex reactions were performed using the primers HLBas (5’ TCGAGCGCGTATGCAATACG 3’) and HLBr (5’ CTACCTTTTTCTACGGGATAACGC 3’) for 16S rDNA bacterial amplification and the primers GAPDH-f (5’ GGTCAGTGGAAGCACAACGA 3’) and GAPDH-r (5’ GAGTACTAAAATGTACCTGAATCCGAAA 3’) for the plant endogenous gene GAPDH. The TaqMan probes HLBp (5’ AGACGGGTGAGTAACGCG 3’) and GAPDH-p (5’ TCCTCTTCGGTGAGAAGCCAGTCGCT 3’) were used to identify the bacterial and citrus amplicons, respectively. Amplifications were performed on the ABI Prism 7500 Sequence Detection System (Applied Biosystems). The reaction mix contained 300 ng DNA, 6.25 μL of 1X TaqMan Universal Master Mix (Applied Biosystems), 216 nM each HLB primer, 270 nM each GAPDH primer, 135 nM each probe, with water added to a final volume of 14 µL. Cycling conditions were 95 °C for 5 min followed by 40 cycles of 95 °C for 30 s and 58 °C for 45 s. The amplifications were performed in duplicate.

The Ct values were used to estimate the bacterial quantification in the plants. The lower the Ct value, the higher the initial concentration of bacteria in the plant, i.e., the greater the genotype’s susceptibility to pathogen attack. According to Boava et al. (2015), samples with Ct values smaller than 34 were considered positive for bacterial multiplication.

All statistical analyses were performed for each trial using linear mixed models with a fixed overall intercept and genotypes as the sole random factor. Both variance components and predicted genotypic values (BLUPs - Best Linear Unbiased Prediction) were estimated simultaneously using lmer from the lme4 package (Bates et al., 2015) implemented in R (R Core Team, 2024). From these models, genetic (Vg) and residual (Ve) variances were extracted to estimate broad-sense heritability defined by ${\text{h}}^{2}=100\times \text{Vg}/(\text{Vg}+\text{Ve})$, as well as the genetic (CVg=100×sqrt(Vg)/µ) and residual (CVr=100×sqrt(Ve)/µ) coefficient of variation, where µ represents the average trial mean. The advantage of using CV is the scale-free property, which allows a direct comparison across trials. In this context, CVr reflects the proportion of non-genetic variation attributable to environmental noise or measurement error, with values close to zero indicating higher experimental precision. Conversely, CVg quantifies the magnitude of available genetic diversity within the population, with higher values preferred for QTL mapping. QTL mapping was performed using composite interval mapping (Zeng, 1994), implemented in the fullsibQTL package (Gazaffi et al., 2014, 2020) on the R platform (R Core Team, 2024). A window size (WS) of 10 cM was used, and 15 cofactors were included in the model to control interference from regions outside the mapping interval and reduce the risk of false-positive detection. The threshold for declaring a QTL was defined from 1000 permutations, considering 5% significance (Churchill and Doerge, 1994). The portion of the phenotypic variation explained by the QTLs (R²) was also estimated.

### 2.4 Candidate genes investigation

To investigate the biological function of the mapped QTL regions, two strategies were employed. The first focused solely on the gene sequences colocalized with the QTL peaks. The second considered an interval of 10 cM upstream and 10 cM downstream of the QTL peak to identify markers with statistical values above the QTL threshold. Afterward, the presence of candidate genes was investigated at each significant marker position, similarly to the first strategy.

These sequences were identified in the Citrus sinensis genome (assembly DVS_A1.0; GCF_022201045.2, available at https://www.ncbi.nlm.nih.gov/datasets/taxonomy/2711) using the nucleotide database and the default BLASTn parameters.

Genes annotated in the C. sinensis genome at the marker position or within a 10 kb flanking region (from 5 kb upstream to 5 kb downstream) were putatively associated with the QTL. The nucleotide sequences of the identified genes were functionally annotated using Blast2GO 6.0.3 (Gotz et al., 2008). A BLASTx analysis was performed with the blastx-fast option and the NCBI-nr database, using Embryophyta (taxa: 3193) as the taxonomy filter, and considering the five best hits with an e-value cutoff of 1 × 10^-5^. Additionally, InterProScan was performed using the EMBL-EBI InterPro web service, while GO mapping and annotation were carried out with default parameters.

## 3 Results

### 3.1 Genetic mapping

Most genotyped loci were monomorphic in the population, rendering them uninformative for mapping studies. Only 23.4% (6466/27690) of DArTseq markers, 7.2% (1251/17482) of DArTseq-derived SNPs, and 10.5% (380/3608) of Infinium SNPs segregated in the population and were available for mapping.

In this scenario, there was a predominance of markers in a 1:1 ratio; consequently, a single map to merge all segregating markers was not viable. Therefore, it was preferable to obtain two maps, one for each parent, similar to the pseudo-testcross (Grattapaglia and Sederoff, 1994), with additional markers segregating in 3:1 and 1:2:1 ratio included on each map (**Table 1**).

**Table 1:** Distribution of molecular markers in the linkage groups obtained by genetic mapping of *C. sunki* and *P. trifoliata*.

Chr^a^	LG^b^	*Citrus sunki*					*Poncirus trifoliata*				
		Length (cM)	Segregation type			Total of markers	Length (cM)	Segregation type			Total of markers
			1:1	3:1	1:2:1			1:1	3:1	1:2:1	
1	1a	204.7	27	0	0	27	112.2	20	3	0	23
	1b	119.3	52	2	0	54	74.0	28	0	0	28
2	2a	487.0	80	2	0	82	376.1	233	5	0	238
	2b	414.7	212	3	0	215	355.8	52	0	0	52
3	3a	572.5	100	0	0	100	412.9	136	1	1	138
	3b	207.5	91	5	2	98	395.4	53	5	1	59
4	4a	411.4	65	0	0	65	367.0	53	1	0	54
	4b	296.0	121	1	0	122	262.0	84	0	0	84
5	5a	230.4	87	2	0	89	281.6	47	1	0	48
	5b	194.5	38	2	0	40	107.7	64	2	0	66
6	6a	317.5	168	3	0	171	332.1	58	0	0	58
	6b	285.8	56	0	0	56	227.1	119	0	0	119
7	7a	433.1	85	0	1	86	599.8	92	0	0	92
	7b	395.2	263	1	1	265	284.6	176	0	0	176
8	8	417.1	138	0	0	138	270.3	33	0	0	33
9	9a	261.5	134	3	0	137	194.0	26	0	0	26
	9b	216.2	37	0	0	37	141.7	46	0	0	46
Total		5464.4				1782	4794.3				1340

^a^Chromosome identification is based on the *Citrus sinensis* reference genome, version DVS_A1.0, available at NCBI (https://www.ncbi.nlm.nih.gov/datasets/taxonomy/2711/). ^b^Linkage group.

**Figure 1:**
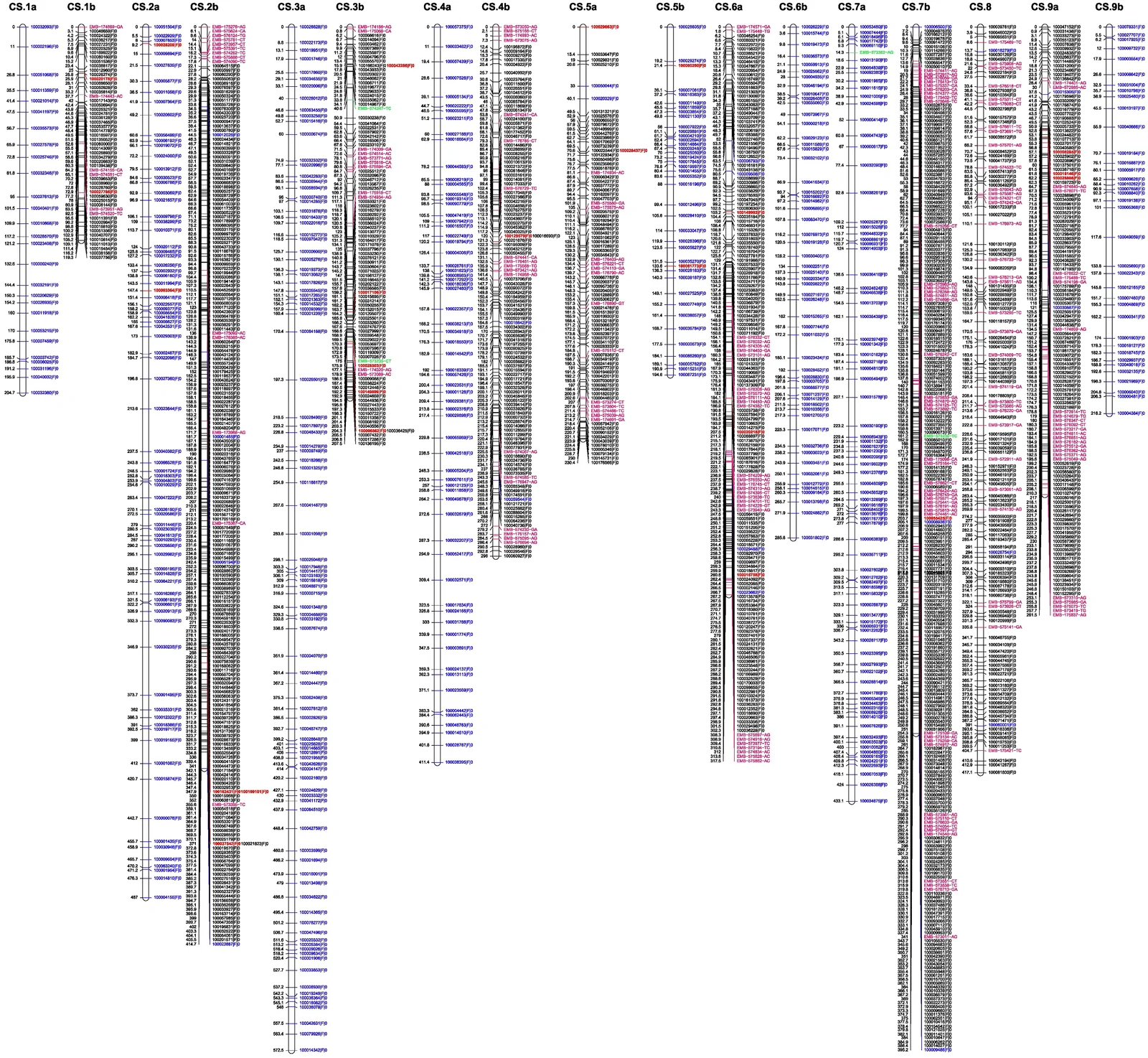
Graphical representation of the *C. sunki* (CS) linkage map obtained with segregation data from DArTseq markers, DArTseq-derived SNPs, and SNPs from the Infinium 65k chip. CS.1a to CS.9b: linkage group identification, representing the nine *Citrus* chromosomes, according to their correspondence based on markers comparison with the *C. sinensis* reference genome (version DVS_A1.0; available at NCBI), using the BLASTn algorithm. Linkage groups with gaps greater than 30 cM were separated into subgroups a and b. Black: DArTseq markers (1:1 segregation); bold red: DArTseq markers (3:1 segregation); blue: DArTseq-derived SNPs (1:1 segregation); magenta: chip-derived SNPs (1:1 segregation); green: chip-derived SNPs (1:2:1 segregation).

**Figure 2:**
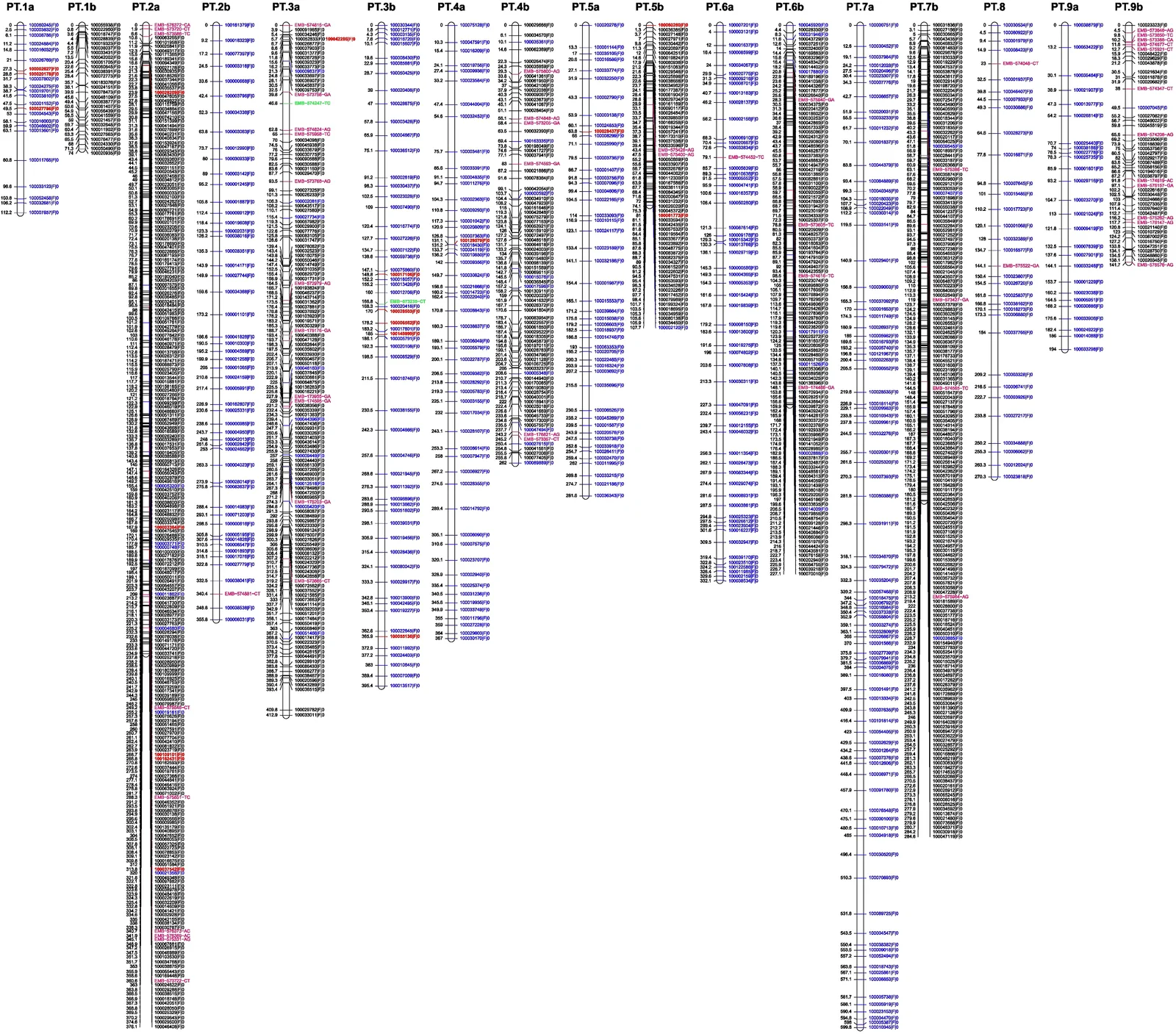
Graphical representation of the *P. trifoliata* (PT) linkage map obtained with segregation data from DArTseq markers, DArTseq-derived SNPs, and SNPs from the Infinium 65k chip. PT.1a to PT.9b: linkage group identification, representing the nine *Citrus* chromosomes, according to their correspondence based on markers comparison with the *C. sinensis* reference genome (version DVS_A1.0; available at NCBI), using the BLASTn algorithm. Linkage groups with gaps greater than 30 cM were separated into subgroups a and b. Black: DArTseq markers (1:1 segregation); bold red: DArTseq markers (3:1 segregation); blue: DArTseq-derived SNPs (1:1 segregation); magenta: chip-derived SNPs (1:1 segregation); green: chip-derived SNPs (1:2:1 segregation).

All nine citrus chromosomes were represented in the genetic maps. The linkage groups obtained from the polymorphisms identified in *C. sunki* and *P. trifoliata* were designated CS and PT, respectively (**Figures 1**, **2**). Linkage groups were numbered 1 to 9, according to their correspondence to *Citrus* chromosomes, identified by BLASTn with the *C. sinensis* reference genome, version DVS_A1.0 (available at NCBI). The mapping analyses generated 17 linkage groups for each parent since the groups corresponding to chromosomes 1, 2, 3, 4, 5, 6, 7, and 9 each showed a gap greater than 30 cM and were separated into subgroups a and b. On the other hand, the loci on chromosome 8 were mapped in a single linkage group.

In groups CS1.a, CS.3a, CS.4a, CS.6b, CS.8, CS.9B, PT.1b, PT.2B, PT4.b, and PT6.a to PT.9b, only 1:1 segregating markers were mapped. Compared with the other linkage groups, the higher occurrence of codominant markers was observed in 3b. In CS.3b, five markers with 3:1 segregation and two with 1:2:1 segregation were mapped. Similarly, PT.3b presented five 3:1 and one 1:2:1 markers.

Overall, the distribution of markers was uneven, suggesting that markers acquired through various technologies tended to sample certain areas of the genome more preferentially. For instance, in the *C. sunki* map, groups CS.1a, CS.3a, CS.4a, CS.6b, and CS.9b were formed solely by DArTseq-derived SNPs (**Figure 1**).

In the *P. trifoliata* map, group PT.1b presents only DArTseq markers with 1:1 segregation, while DArTseq-derived SNP entirely forms groups PT.7a and PT.9a (**Figure 2**).

The CS linkage map comprised 1872 markers spanning a total distance of 5464.4 cM, with individual groups ranging from 119.3 cM (CS.1b) to 572.5 cM (CS.3a) (**Table 1**). The map showed an average marker density of 3.1 cM, with CS.7b identified as the most saturated linkage group (265 markers over 395.2 cM) and CS.1a as the least saturated (27 markers over 204.7 cM) (**Table 1**; **Supplementary Figure 1**). Marker ordering generally deviated from physical positions, except for CS.8, which exhibited notable synteny with chromosome 8 of *C. sinensis*. In parallel, the PT map integrated 1340 markers across 4794.0 cM, with group lengths ranging from 74.0 cM (PT.1b) to 599.8 cM (PT.7a) and maintaining a comparable average density of 3.6 cM (**Table 1**; **Supplementary Figure 2**).

A detailed assessment of both maps revealed a non-uniform distribution of markers across the chromosomes, influenced by technology-dependent clustering preferences. In the CS map (**Figure 1**), eight linkage groups (1a, 2a, 3a, 4a, 5b, 6b, 7a, and 9b) were primarily composed of DArTseq-derived SNPs, covering a total of 2805.2 cM with a lower average density of 5.69 cM per marker. In contrast, nine linkage groups (1b, 2b, 3b, 4b, 5a, 6a, 7b, 8, and 9a) were mainly defined by DArTseq markers, spanning 2659.2 cM and exhibiting a substantially higher density of 2.06 cM per marker.

A minor symmetrical trend was observed in the PT map (**Figure 2**), where nine groups were predominantly composed of DArTseq-derived SNPs (1a, 2b, 3b, 4b, 5b, 6a, 7a, 8, and 9a), covering 2908.2 cM with an average density of 6.54 cM per marker. In comparison, eight groups (1b, 2a, 3a, 4b, 5b, 6b, 7b, and 9b) were primarily formed by DArTseq markers, covering 1886.1 cM with an average density of 2.11 cM per marker.

Overall, DArTseq markers demonstrated greater coverage efficiency than DArTseq-derived SNPs, consistently providing highly saturated intervals with significantly lower inter-marker distances across both maps.

### 3.2 Phenotyping and statistical analyses

The mapping population was phenotyped in four experiments, genetically characterized in **Table 2**. The average Ct ranged from 24.4 (Field-2014 and Greenhouse-2021) to 29.0 (Greenhouse-2009). The minimum Ct value, which is indicative of susceptibility, was 20.0 (Greenhouse-2021), 20.8 (Field-2018), 23.3 (Greenhouse-2009), and 14.7 (Field-2014). It is worth mentioning that in Field-2014, two other Ct values (19.9 and 19.8) were lower than the minimum Ct observed in the other three experiments, indicating greater susceptibility to HLB. The maximum Ct values ranged from 32.2 (Field-2014) to 38.9 (Field-2018).

**Table 2:** Statistical and genetic parameters estimated from the Ct obtained in the four experiments evaluating citrus response to HLB.

Experiment	minCT^a^	maxCT^b^	avgCT^c^	Vg^d^	CVg^e^	Vr^f^	CVr^g^	h^2h^	Evaluated genotypes
Field-2014	14.7	32.2	24.4	20.34	18.46	7.86	11.48	72.12	66
Field-2018	20.8	38.9	28.0	27.81	18.81	6.24	8.91	81.67	265
Greenhouse-2009	23.3	37.0	29.0	15.41	13.52	9.34	10.53	62.28	90
Greenhouse-2021	20.0	36.0	24.4	21.46	18.97	5.11	9.26	80.74	71

^a^Minimal Ct value; ^b^Maximum Ct value; ^c^Average Ct value; ^d^Genetic variance; ^e^Coefficient of variation for genotypic variance obtained 100×sqrt(Vg)/avgCT; ^f^Residual variance; ^g^Coefficient of variation of the residue obtained by 100×sqrt(Vr)/avgCT; ^h^Heritability obtained by 100×Vg/(Vg+Ve).

Regarding the residual coefficient of variation (CVr), the lowest values were observed in Field-2018 (8.91%) and Greenhouse-2021 (9.26%), which represent the most recent trials chronologically. Conversely, the highest CVr values occurred in Greenhouse-2009 (10.53%) and Field-2014 (11.48%).

The broad-sense heritability was moderate to high across all environments, with Greenhouse-2009 yielding the lowest estimate (62.28%), whereas the remaining trials exceeded 70%, with Field-2018 and Greenhouse-2021 surpassing 80%. The same pattern can be observed for CVg (**Table 2**), confirming the robust genetic variability within each trial. While the four experiments exhibited similar overarching genetic patterns, further investigations were conducted to assess genotypic trends across trials. In this work, a partially overlapping genotyping strategy was adopted to accommodate the logistics of screening a large full-sib population over time. Out of the total population (278 genotypes), specific cohorts were characterized in Field-2014 (n = 66), Greenhouse-2021 (n = 71), and Greenhouse-2009 (n = 90), while Field-2018 provided an extensive evaluation with 265 genotypes. Given this partitioned structure, pairwise comparisons of individual genotype performance focus on distinct shared cohorts. Within these specific overlapping subsets, the low to negative correlation values ranged from -0.21 (Field-2014 vs. Greenhouse-2021; 9 genotypes) and -0.37 (Field-2014 vs. Greenhouse-2009; 16 genotypes) to 0.19 (Field-2018 vs. Greenhouse-2009; 89 genotypes). Importantly, these low absolute correlations underscore the presence of a complex Genotype-by-Environment (G×E) interaction, further highlighting that conducting four independent analyses provides a more reliable framework to demonstrate the repeatability of our QTL mapping results.

### 3.3 QTL mapping

A total of 44 QTLs related to citrus response to HLB were identified (**Supplementary Figures 3**–**11**), 25 of which were identified from the CS map (**Table 3**) and the other 19 from the PT map (**Table 4**).

**Table 3:** QTLs related to citrandarin hybrids’ response to HLB identified from the *Citrus sunki*’s genetic map (CS).

QTL name	LG	Marker	Position (cM)	$-\log_{10}pvalue$	R^2^ (%)	Effects			Trial
						CS^a^	PT^b^	Dominance^c^	
CS-HLB1	CS1.b	100024322|F|0	4.21	11.31	29.89	-2.41	0.77	1.80	Field 2014
CS-HLB2	CS.3b	100008616|F|0	6.21	10.94	15.87	1.58	2.09	0.02	
CS-HLB3	CS.4b	100195009|F|0	122.35	11.12	19.27	2.30	0.20	NA	
CS-HLB4	CS.6a	100024092|F|0	262.41	5.77	8.04	-0.39	-1.63	NA	
CS-HLB5	CS.7a	100002095|F|0	240.58	16.40	29.86	-2.54	NA	NA	
CS-HLB6	CS.8	100017233|F|0	100.11	6.16	6.74	-1.38	NA	NA	
					68.97				
CS-HLB7	CS.3a	100016818|F|0	308.96	4.80	3.83	-2.17	NA	NA	Field 2018
CS-HLB8	CS.3b	100012446|F|0	190.54	8.48	8.77	1.21	-1.82	0.67	
CS-HLB9	CS.4a	100029898|F|0	217.38	10.70	9.52	2.18	NA	NA	
CS-HLB10	CS.4b	100026458|F|0	152.33	6.26	3.45	-0.65	3.14	NA	
CS-HLB11	CS.5a	100033647|F|0	15.37	6.27	5.51	-0.47	-2.90	NA	
CS-HLB12	CS.6a	100046768|F|0	282.73	5.27	4.76	-1.53	-2.64	NA	
CS-HLB13	CS.8	100027022|F|0	105.10	4.40	3.42	-1.78	NA	NA	
					34.06				
CS-HLB14	CS.3b	100025512|F|0	84.72	5.27	5.46	0.99	1.82	0.15	Greenhouse 2009
CS-HLB15	CS.4b	100016642|F|0	164.59	12.89	23.25	2.95	-0.67	NA	
CS-HLB16	CS.4b	100037597|F|0	185.46	8.76	11.45	-2.01	NA	NA	
CS-HLB17	CS.5a	EMB-176890-GT	157.32	9.98	13.84	-1.56	NA	NA	
CS-HLB18	CS5.b	100086260|F|0	184.06	5.75	11.19	0.95	-1.35	NA	
CS-HLB19	CS.7a	100005494|F|0	196.86	6.81	8.57	1.69	NA	NA	
CS-HLB20	CS.7a	100002687|F|0	323.32	5.75	7.23	1.18	NA	NA	
CS-HLB21	CS.7b	EMB-576621-CT	185.56	7.83	16.23	-0.11	-1.51	1.23	
					72.01				
CS-HLB22	CS.2a	100064221|F|0	310.22	12.69	21.88	3.17	NA	NA	Greenhouse 2021
CS-HLB23	CS.2a	100014810|F|0	476.33	14.63	27.19	-3.33	NA	NA	
CS-HLB24	CS.3a	100062408|F|0	375.34	6.83	9.10	1.49	NA	NA	
CS-HLB25	CS.3a	100016001|F|0	473.89	6.61	8.71	-1.96	NA	NA	
					39.65				

^a^*Citrus sunki* additive effect. ^b^*Poncirus trifoliata* additive effect. ^c^Dominance effect. Phenotypic evaluations were conducted under field conditions (2014 and 2018) and greenhouse (2009 and 2021).

**Table 4:** QTLs related to citrandarin hybrids’ response to HLB were identified from the *Poncirus trifoliata*’s genetic map (PT).

QTL name	LG	Marker	Position (cM)	$-\log_{10}pvalue$	R^2^ (%)	Effects			Trial
						CS^a^	PT^b^	Dominance^c^	
PT-HLB1	PT.1a	100060245|F|0	0.00	6.90	1.07	0.25	0.19	1.74	Field 2014
PT-HLB2	PT.2a	100011862|F|0	208.99	5.41	0.72	-0.13	0.49	1.84	
PT-HLB3	PT.3a	100037903|F|0	8.61	8.28	14.80	0.88	1.55	0.82	
PT-HLB4	PT.4a	100006090|F|0	305.11	5.32	5.39	NA	-1.21	NA	
PT-HLB5	PT.7b	100051796|F|0	157.43	6.80	7.24	NA	1.33	NA	
					31.05				
PT-HLB6	PT.3b	100007122|F|0	134.66	18.55	1.28	-5.50	0.17	-0.09	Field 2018
PT-HLB7	PT.4b	100015076|F|0	145.49	6.09	6.03	NA	-1.34	NA	
PT-HLB8	PT.6a	100001637|F|0	12.01	7.91	8.31	NA	-1.82	NA	
PT-HLB9	PT.7a	100011232|F|0	61.69	4.27	3.50	NA	-1.05	NA	
					17.20				
PT-HLB10	PT.1a	100008786|F|0	6.13	5.36	1.57	-0.27	-0.66	1.52	Greenhouse 2009
PT-HLB11	PT.2a	EMB-575546-CT	249.25	5.29	7.90	-0.52	-0.94	-1.46	
PT-HLB12	PT.2a	100042410|F|0	262.35	7.39	17.25	-1.06	1.40	2.10	
PT-HLB13	PT.4a	100005783|F|0	219.28	4.80	6.22	NA	0.95	NA	
PT-HLB14	PT.5a	100003736|F|0	79.46	7.38	7.73	0.55	1.67	NA	
PT-HLB15	PT.5a	100001987|F|0	154.40	11.81	16.17	NA	-1.90	NA	
					40.28				
PT-HLB16	PT.2b	100161379|F|0	0.00	9.00	14.97	NA	-1.95	NA	Greenhouse 2021
PT-HLB17	PT.3a	EMB-574815-GA	0.00	13.84	32.88	-2.10	2.04	-1.48	
PT-HLB18	PT.3a	100017464|F|0	356.13	5.65	7.92	NA	-1.48	NA	
PT-HLB19	PT.9b	EMB-576157-GA	97.11	5.89	8.17	NA	-1.48	NA	
					54.81				

^a^*Citrus sunki* additive effect. ^b^*Poncirus trifoliata* additive effect. ^c^Dominance effect. Phenotypic evaluations were conducted under field conditions (2014 and 2018) and greenhouse (2009 and 2021).

Based on field evaluations, six QTLs (CS-HLB1 to CS-HLB6) were mapped in 2014 for the CS.1b, CS.3b, CS.4b, CS.6a, CS.7a, and CS.8 groups, respectively. They jointly explain 68.97% of the phenotypic variation (R²), with individual R² values ranging from 6.74% (CS-HLB6) to 29.89% (CS-HLB1), located in the CS.8 and CS.1a groups, respectively (**Table 3**).

The phenotyping data from 2018 allowed the identification of seven QTLs (CS-HLB7 to CS-HLB13) in the CS.3a, CS.3b, CS.4a, CS.4b, CS.5a, CS.6a, and CS.8 groups, explaining approximately 34% of the phenotypic variation. As observed in 2014, the QTL with the lowest R² value (3.42%) was identified in group CS.8. Conversely, the highest percentage of phenotypic variation was explained by CS-HLB9, located in group CS.4a (**Table 3**).

The same experiments identified fewer QTLs from the *P. trifoliata* (PT) map; in 2014, five QTLs (PT-HLB1 to PT-HLB5) mapped in groups PT.1a, PT.2a, PT.3a, PT.4a, and PT.7b, explaining 31% of the phenotypic variation. Of particular note were the QTLs PT-HLB2 in group PT.2a and PT-HLB3 in group PT.3a, with the lowest (0.72%) and highest (14.80%) R2 values, respectively (**Table 4**).

Four QTLs (PT-HLB6 to PT-HLB9) were mapped in 2018, in the groups PT.3b, PT.4b, PT6.a, and PT.7a (**Table 4**), accounting for 17.20% of the phenotypic variation. PT-HLB6, mapped in group PT.3b, presented the lowest individual R² value (1.57%), while the highest R² (8.31%) was represented by PT-HLB8, located in group PT.6a.

In contrast, the HLB response evaluated under greenhouse conditions allowed the identification of 12 QTLs on the CS map. Eight QTLs were mapped using phenotyping data collected in 2009, one in the CS.3b group (CS-HLB1), two in CS.4b (CS-HLB15 and CS-HLB16), one in CS.5a (CS-HLB17), one in CS.5b (CS-HLB18), two in CS.7a (CS-HLB19 and CS-HLB20), and one in CS.7b (CS-HLB21). Seventy-two percent of the phenotypic variation was explained by these QTLs, with individual R² ranging from 5.46% for CS-HLB14, located in CS.3b, to 23.25% for CS-HLB15, mapped in CS.4b (**Table 3**).

In 2021, CS-HLB22 and CS-HLB23 were mapped in group CS.2a, and CS-HLB24 and CS-HLB25 in CS.3a. The phenotypic variation explained by these QTLs was 39.65%, with the highest R² value (27.19%) corresponding to CS-HLB23 and the lowest (8.71%) explained by CS-HLB25 (**Table 3**).

Regarding the *P. trifoliata* map, six QTLs associated with greenhouse evaluations conducted in 2009 were identified (**Table 4**). One QTL was mapped in PT.1a (PT-HLB10), two in PT.2a (PT-HLB11 and PT-HLB12), one in PT.4a (PT-HL13), and two in PT.5a (PT-HLB14 and PT-HLB15). Approximately 40% of the phenotypic variation was explained by these QTLs, with individual R² ranging from 6.22% (PT-HL13) to 17.25% (PT-HLB12). In 2021, one QTL was identified in PT.2b (HLB-16), two in PT3.a (PT-HLB17 and PT-HLB18), and one in PT.9b (PT-HLB19). Together, these 4 QTLs explained 54.81% of the phenotypic variation, and individual R² values ranged from 5.65% (PT-HLB18) to 13.88% (PT-HLB17).

### 3.4 Candidate genes investigation

The QTL biological rules were investigated by functional annotation of gene sequences in *the C. sinensis* genome at specific sites corresponding to the molecular markers. The first investigation focused on genes at the specific QTL position to obtain an initial candidate gene list, considering a 10 kb flanking region (5 kb upstream to 5 kb downstream) around the molecular marker associated with the QTL (**Supplementary Table 1**).

Of the 25 QTLs detected in the CS genetic map, 17 (CS-HLB2, CS-HLB5, CS-HLB7, CS-HLB8, CS-HLB9, CS-HLB11, CS-HLB12, CS-HLB15, CS-HLB16, CS-HLB17, CS-HLB18, CS-HLB19, CS-HLB20, CS-HLB21, CS-HLB22, CS-HLB23, CS-HLB25) were positioned at gene sites (**Supplementary Table 1**).

The QTL CS-HLB5, detected in the CS.7a linkage group from field evaluation in 2014, was associated with a probable disease resistance protein, similar to At5g63020. In a field evaluation carried out in 2018, QTLs were identified associated with a TMV resistance protein N-like (CS-HLB8), a GEM-like protein 4 (CS-HLB9), and a SH3 domain-containing protein 1-like (CS-HLB11), which were mapped on CS.3b, CS.4a, and CS.5a linkage groups, respectively (**Supplementary Table 1**).

Evaluations performed in greenhouse conditions in 2009 detected QTLs related to an integrin-linked protein kinase 1 (CS-HLB15) and a protein FLX-like 3 (CS-HLB16) in the linkage group CS.4b. An ABC transporter B family member 13-like (CS-HLB18) and a protein transport protein SEC13 homolog B-like (CS-HLB21) were mapped in CS.5b and CS.7b, respectively (**Supplementary Table 1**).

The QTLs identified in greenhouse conditions in 2021 were associated with a pentatricopeptide repeat-containing protein (At5g46460), a mitochondrial protein (CS-HLB23), and a transcription factor DYT1 (CS-HLB22) mapped in CS.2a, as well as a general transcription and DNA repair factor IIH helicase subunit XPD (CS-HLB25) identified in CS.3a linkage group (**Supplementary Table 1**).

The other six QTLs (CS-HLB2, CS-HLB7, CS-HLB12, CS-HLB17, CS-HLB19, and CS-HLB20) were associated with uncharacterized loci that could be classified using Blast2GO. CS-HLB2, identified in linkage group CS.3b through a field evaluation conducted in 2014, is linked to a hypothetical protein containing a hydrophobic non-cytoplasmic transmembrane domain.

CS-HLB7 and CS-HLB12, identified in the CS.3a and CS.6a linkage groups from field experimental data gathered in 2018, were categorized as a WD repeat domain protein and an ABC transporter G family member 43-like isoform X1, respectively (**Supplementary Table 1**).

The QTLs identified from greenhouse evaluation in 2009 CS-HLB17 (LG CS.5a), CS-HLB19 (LG CS.7a), and CS-HLB20 (LG CS.7a) were annotated as an uncharacterized protein (LOC102626479), a LEA 2 domain-containing protein, and a pyridoxamine 5’-phosphate oxidase family protein, respectively (**Supplementary Table 1**).

Among the other eight QTLs detected in CS (CS-HLB1, CS-HLB3, CS-HLB4, CS-HLB10, CS-HLB14, CS-HLB24), six are less than 5 kb upstream or downstream of predicted gene sequences in the *C. sinensis* genome. From field evaluation in 2014, CS-HLB1 (LG CS.1b) and CS-HLB3 (LG CS.4b) were both annotated as reverse transcriptase domain-containing proteins, and CS-HLB4 (LG CS.6a) was classified as an uncharacterized hypothetical protein. A retrovirus-related polyprotein from the transposon RE1 was associated with CS-HLB10 (LG CS.4b), which was noticed in field evaluations in 2018 (**Supplementary Table 1**). A protein transport protein SEC16B homolog isoform X1 and a NAC domain-containing protein 83 were related to CS-HLB14 (LG CS.3b) and CS-HLB24 (LG CS.3a), observed in greenhouse conditions in 2009 and 2021, respectively (**Supplementary Table 1**).

The other two QTLs (CS-HLB6 and CS-HLB13) mapped to LG CS.8, based on field evaluations in 2014 and 2018, could not be functionally annotated, as no similarity was found between the nucleotide sequences of the molecular markers and the *C. sinensis* reference genome (**Supplementary Table 1**).

The second strategy used to investigate the putative function of genomic regions responsible for the QTLs’ effects considered all molecular markers significantly associated with HLB response and mapped within a maximum distance of 10 cM upstream or downstream of the QTL peak in order to broaden the possibilities of finding candidate genes related to HLB (**Supplementary Table 2**).

Out of the 25 QTLs identified in CS, 17 (CS-HLB1, CS-HLB2, CS-HLB3, CS-HLB4, CS-HLB5, CS-HLB8, CS-HLB9, CS-HLB10, CS-HLB11, CS-HLB12, CS-HLB15, CS-HLB16, CS-HLB17, CS-HLB21, CS-HLB22, CS-HLB23, CS-HLB24) showed significant markers located in the regions next to the QTL peaks (**Supplementary Table 2**).

In the CS-HLB1 region, the molecular markers EMB-174869-GA, 100046659|F|0, 100038217|F|0, and 100027231|F|0 have been identified as a hypothetical protein, a peroxygenase 1, a splicing factor U2af large subunit B, and a 4-coumarate-CoA ligase-like 9, respectively (see **Supplementary Table 2**).

The significant markers CS-HLB2 include EMB-175068-CA, 100014094|F|0, 100003462|F|0, and 100007389|F|0, which were identified as a Dof zinc finger protein DOF4.6, an embryogenesis-associated protein EMB8, a transfer RNA for cysteine, and a protein from the pentatricopeptide repeat (PPR) superfamily (see **Supplementary Table 2**).

The QTL CS-HLB3, located on linkage group CS.4b, is associated with the coding regions of the MDIS1-interacting receptor kinase 2 (MIK2), a hypothetical protein, a protein containing a Str synth domain, and the transcriptional regulator ATRX, which are captured by the markers 100129579|F|0, 100016690|F|0, 100050455|F|0, and EMB-574441-CA, respectively. Additionally, the protein H3 from the glycine cleavage system was identified, linked to the markers 100201676|F|0 and 100043212|F|0 (see **Supplementary Table 2**).

CS-HLB4 was significantly associated with the Ras-related protein RabE1a, the INO80 complex subunit D, and a BED-type domain-containing protein, identified by markers 100018817|F|0, 100016758|F|0, and 100052085|F|0, respectively. Two other markers, 100013578|F|0 and 100016734|F|0, were identified in the coding region of a retrovirus-related pol polyprotein from transposon RE1 (**Supplementary Table 2**).

Alongside the 100002095|F|0 marker, which is colocalized with the CS-HLB5 QTL peak, three additional markers displayed a significant association with this QTL. Two of these markers, 100007627|F|0 and 100001230|F|0, were not found in the *C. sinensis* reference genome; hence, their potential functions remain undetermined. Conversely, the third marker (100019602|F|0) was located within the coding region of a glucan endo-1, 3-beta-D-glucosidase (**Supplementary Table 2**).

A total of six genes involved in stress response have been linked to the QTL CS-HLB8, which is located on LG CS.3b. These genes include a pleiotropic drug resistance protein 1-like (PDR1-like), the acetolactate synthase (ALS) small subunit 2, a transmembrane protein, a disease resistance protein RPV1-like, a SUN domain-containing protein 4-like, and a disease resistance-like protein DSC1. Each of these genes was identified using the following molecular markers: EMB-573089-AC, 100009088|F|0, 100038224|F|0, 100149899|F|0, 100024659|F|0, and 100044936|F|0 (as shown in **Supplementary Table 2**).

The QTL CS-HLB9 has been linked to the MAP3Kε1 gene, and a plant Kelch-containing F-box protein, as determined by markers 100029381|F|0 and 100002376|F|0, respectively (**Supplementary Table 2**).

Five molecular markers showed a significant association with the QTL CS-HLB10. Specifically, markers 100043028|F|0, 100090072|F|0, and 100004242|F|0 were annotated as a probable cytokinin riboside 5’-monophosphate phosphoribohydrolase LOGL10, an F-box/LRR-repeat protein, and a retrovirus-related Pol polyprotein, respectively. Furthermore, two additional markers were recognized within this QTL: 100073706|F|0, which does not match any known *C. sinensis* gene, and 100184959|F|0, which is identified as a hypothetical protein (**Supplementary Table 2**).

The molecular marker 100029831|F|0 demonstrated a significant correlation with the QTL CS-HLB11; however, it could not be functionally annotated since it was not found in the *C. sinensis* genome (**Supplementary Table 2**).

The CS-HLB12 QTL was linked to marker 100048506|F|0, found within the coding region of a putative LRR receptor-like serine/threonine-protein kinase, as well as to markers 100024131|F|0 and 100032821|F|0, which have not been identified in the referenced genome (**Supplementary Table 2**).

CS-HLB15 has been linked to epidermal patterning factor-like protein 9 and the putative inactive cysteine synthase 2 isoform X3, which were identified by markers 100034302|F|0 and 100026943|F|0, respectively (**Supplementary Table 2**).

The QTL CS-HLB15 was associated with epidermal patterning factor-like protein 9 and the putative inactive cysteine synthase 2 isoform X3, which were identified by markers 100034302|F|0 and 100026943|F|0, respectively (**Supplementary Table 2**).

The only marker 100055682|F|0 that demonstrated a significant correlation with the CS-HLB16 QTL was found within the coding region of a protein that possesses a B3 domain (**Supplementary Table 2**). A total of five markers showed a significant association with QTL CS-H17. Markers 100020405|F|0, 100012889|F|0, 100053062|F|0, and 100015139|F|0 were found in coding regions of a vacuolar protein sorting-associated protein 45-like, a putative serine/threonine-protein kinase At1g54610, an ethylene-responsive transcription factor ABR1, and an oxidoreductase At4g09670, respectively. In contrast, marker 100021476|F|0 was not detectable in the reference genome used for gene annotation (**Supplementary Table 2**).

The CS-HLB21 QTL showed a correlation with genes that encode the JINGUBANG protein, a protein containing the RECA 2 domain, as well as a protein with a CCHC-type domain, identified by markers 100009168|F|0, 100032236|F|0, and 100006589|F|0. Additionally, the marker 100057874|F|0, which is not found in the *C. sinensis* genome, was also linked to this QTL (**Supplementary Table 2**).

Within the CS-HLB23 QTL interval region, only marker 100004155|F|0 demonstrated statistical significance; however, its potential function could not be ascertained. It is situated in the coding region of a hypothetical protein (**Supplementary Table 2**).

Genes that code for cysteine-rich receptor-like protein kinase 10 and mitochondrial isoform X2 of ornithine aminotransferase, identified by markers 100014828|F|0 and 100016266|F|0, were linked to the QTL CS-HLB23.

Ultimately, CS-HLB24 was linked to an extensin-2-like protein, indicated by marker 100024447|F|0.

Considering the 19 QTLs detected in the PT genetic map, 16 (PT-HLB1, PT-HLB2, PT-HLB3, PT-HLB4, PT-HLB7, PT-HLB8, PT-HLB9, PT-HLB10, PT-HLB11, PT-HLB12, PT-HLB13, PT-HLB15, PT-HLB16, PT-HLB17, PT-HLB18, and PT-HLB19) were positioned at gene sites (**Supplementary Table 3**). The QTLs identified through field evaluation carried out in 2014, PT-HLB1, PT-HLB2, PT-HLB3, and PT-HLB4, were respectively annotated as a pentatricopeptide repeat-containing protein, a brassinosteroid-related acyltransferase 1-like, a hypothetical protein with transcription coregulator activity, and a transcription factor MYB124/MYB88 (**Supplementary Table 3**). The QTLs PT-HLB7 (LG PT.4b), PT-HLB8 (LG PT.6a), and PT-HLB9 (LG PT.7a) detected from field evaluation in 2018 were associated with a dihydroorotate dehydrogenase (quinone) mitochondrial, a protein CLP1 homolog, and a splicing factor U2af large subunit B (**Supplementary Table 3**). At greenhouse conditions in 2009, were detected the QTLs PT-HLB10 (LG PT.1a), PT-HLB11 (LG PT.2a), PT-HLB12 (LG PT.2a), PT-HLB13 (LG PT.4a), and PT-HLB15 (LG PT.5a) associated with the splicing factor U2af large subunit B, a pyruvate kinase, the photosystem I reaction center subunit III chloroplastic, a uncharacterized protein, and the thioredoxin Y1 chloroplastic (**Supplementary Table 3**). The PT-HLB16, mapped in LG PT2.b through evaluation at the greenhouse in 2021, is represented by the DArTseq-derived marker 100161379|F|0, which shows three hits in the *C. sinensis* chromosome 2, identified by the loci LOC107174216, LOC102607325, and LOC102615475, that represent peroxidase (**Supplementary Table 3**). The QTLs PT-HLB17 (LG PT.3a), PT-HLB18 (LG PT.3a), and PT-HLB19 (LG PT.9b), also identified at the greenhouse in 2021, were associated with a methionine gamma-lyase, a general transcription and DNA repair factor IIH helicase subunit XPD, and a thioredoxin reductase NTRC, respectively.

Finally, the QTLs PT-HLB5 (LG PT.7b), PT-HLB6 (LG PT.3b), and PT-HLB14 (LG PT.5a), respectively identified in the field evaluations in 2014 and 2018, and the greenhouse experiment in 2009, did not show similarity to the *C. sinensis* genome and could not be functionally annotated (**Supplementary Table 3**).

Using the second functional annotation approach, which considers all statistically significant molecular markers within 10 cM of the QTL peak, candidate genes were identified in 11 (PT-HLB1, PT-HLB2, PT-HLB3, PT-HLB4, PT-HLB5, PT-HLB7, PT-HLB8, PT-HLB12, PT-HLB14, PT-HLB17, PT-HLB18) out of the 19 QTLs mapped in PT (**Supplementary Table 4**).

In the QTL PT-HLB1, two proteins were identified: the U2af large subunit B, a splicing factor, and Sec20, a vesicle transport protein, associated with markers 100008786|F|0 and 100003832|F|0, respectively (**Supplementary Table 4**).

PT-HLB2 was associated with a probable reverse transcriptase, identified by marker 100023687|F|0, as well as a segment that is absent in the C. sinensis reference genome, marked by 100041730|F|0 (**Supplementary Table 4**).

The QTL PT-HB3 was linked to four proteins that may play a role in stress response: a BRUTUS-like protein (BTS); a LURP-one-related 4 (LOR4) protein; a COP1-interactive mitochondrial protein; and a substrate carrier family protein. These potential regions were identified through markers 100033773|F|0, 100026728|F|0, 100026877|F|0, and 100031377|F|0, respectively. Marker 100039323|F|0, which was also significantly related to this QTL, was absent from the reference genome (**Supplementary Table 4**).

In the PT-HLB4 interval, only the marker 100007575|F|0 exhibited a statistically significant link with this QTL. This marker was identified within the coding region of the BPA1 gene (Binding Partner of ACD11-1), which is known for its role in plant defense mechanisms (**Supplementary Table 4**).

The molecular marker 100043095|F|0, located 160 cM from LG PT.7b and showing a significant association with the PT-HLB5 QTL, was identified at four sites on chromosome 7 of *C. sinensis*, spanning approximately 65.6 Mb, suggesting multiple copies and gene clustering. Each of the four regions aligns with the resistance gene locus, similar to At5g63020. The sequence of 100043095|F|0 aligns to two positions of the LOC102626337 locus and to one position each of the LOC127903670 and LOC102625482 loci. Markers 100035405|F|0 and 100167848|F|0 were also associated with PT-HLB5; however, no annealing sequences were found for these within the genome used for annotation (**Supplementary Table 4**).

The genes that code for a probable protein arginine N-methyltransferase 6, nucleolin 2 protein, and chromatin remodeling 20 (CHR20), identified by markers 100036845|F|0, 100069611|F|0, and 100151589|F|0, were linked to the QTL PT-HLB7 (**Supplementary Table 4**).

Two genes were associated with the PT-HLB8 QTL: the one encoding Acyl transport protein 2 (ACP2), sampled by marker 100003131|F|0, and the one encoding Biotin carboxyl transport protein (BCCP), identified by marker 100008599|F|0 (**Supplementary Table 4**).

In the PT-HLB12 QTL mapping interval, we identified eight markers that exhibited significant correlations. Of these, four markers (100079987|F|0, 100027970|F|0, 100081822|F|0, and 100023719|F|0) were not found in the reference genome. The remaining four markers, 100023194|F|0, 100077704|F|0, 100109101|F|0, and 100162431|F|0, were linked to peroxidase 7, Ras-related protein RABF2b, helicase-like transcription factor CHR28, and cysteine-rich receptor-like protein kinase 10, respectively (**Supplementary Table 4**).

Besides marker 100003736|F|0, which indicates the QTL peak, the 100025990|F|0 marker was identified in PT-HLB14; nevertheless, neither of them could be functionally annotated due to their lack of correspondence with the referenced genome (**Supplementary Table 4**).

In QTL PT-HLB17, markers 100091965|F|0 and 100031377|F|0 were identified as being associated with N6-adenosine methyltransferase MT-A70-like and a protein from the mitochondrial substrate transporter family, respectively. In contrast, marker 100037903|F|0 is located within a coding region of a hypothetical protein (**Supplementary Table 4**).

The final QTL mapped in PT, which presented significant markers in its mapping interval, was PT-HLB18. This QTL was significantly associated with a probable FZO-type, chloroplastic transmembrane GTPase, identified by marker 100050419|F|0 (**Supplementary Table 4**).

## 4 Discussion

Understanding the genetic control of HLB resistance remains a challenge due to its quantitative trait nature and the difficulty in defining a standard commercial resistant genotype. To our knowledge, fully resistant genotypes to HLB have not yet been reported. However, several studies have demonstrated tolerance in citrus-related genera (Ramadugu et al., 2016), including *Poncirus* (Folimonova et al., 2009; Boscariol-Camargo et al., 2010; Albrecht and Bowman, 2012; Boava et al., 2017; Huang et al., 2018, 2023). To shed light on the HLB genetic architecture, QTL mapping enables us to identify regions of the citrus genome related to HLB, and candidate genes can be pinpointed by investigating these loci. In this scenario, an inter-genus genetic map was obtained by crossing *C. sunki* × *P. trifoliata* and combining DArTseq, DArTseq-derived SNPs, and SNPs from the Infinium 65k chip, followed by QTL mapping and candidate gene annotation for the mapped regions.

### 4.1 Polymorphism and linkage maps

In a full-sib cross, Wu et al. (2002a) reported all possible segregation patterns for molecular markers in a diploid context. Here, in this inter-genus cross, most sampled loci were monomorphic, with a low rate of segregating markers even with high-throughput markers. Considering only markers on the genetic map, 3140 markers were classified into segregation patterns (1:2:1, 3:1, and 1:1), with only 0.97% (30/3104) showing informative segregation patterns for both parents simultaneously (1:2:1 or 3:1); this low percentage was an issue. Similar results were reported by Curtolo et al. (2017) using only DArTseq markers to genotype the same *C. sunki* × *P. trifoliata* population. In the construction of integrated genetic maps from the F1 generation, a predominance of molecular markers segregating in a 1:1 ratio is frequently observed, at the expense of more informative markers. Contextualizing, Balsalobre et al. (2017) obtained an integrated genetic map in a sugarcane full-sib cross using 996 single-dose markers, in which 724 (73%) segregated in a 1:1 fashion and 269 (27%) segregated in either a 1:2:1 or 3:1 fashion. Rosa et al. (2018) obtained a genetic map for rubber trees, reporting that 178 of 362 (48.9%) were informative for both parents (145 showed a 1:1:1:1 fashion and 33 showed a 1:2:1 fashion), and 184 markers (50.1%) showed a 1:1 segregation pattern. In both situations, the parents shared the same species level.

Routinely, the pseudo-testcross strategy (Grattapaglia and Sederoff, 1994) was the main approach in a full-sib cross, in which only markers segregating in a 1:1 fashion were used for genetic mapping, providing two genetic maps, one for each parent. Later, Wu et al. (2002b) proposed a multipoint approach using Hidden Markov Models to obtain a single map considering more informative markers; this methodology was implemented in OneMap software (Margarido et al., 2007; Taniguti et al., 2022) and was applied to obtain our map. However, the methodology is maximized when markers segregating in both parents are frequent; given the lack of 1:1:1:1 markers and the limited number of 1:2:1 and 3:1 segregation rates, the strategy of presenting two maps was maintained, with the addition of markers with 1:2:1 and 3:1 in each map.

These marker segregation distributions emphasize the large genetic distance between the parents, as they belong to different genera, indicating that a shared polymorphism would be very conserved and rare in this dataset for the dominant, as cited by Curtolo et al. (2017), and co-dominant, as verified here. Consequently, it makes it challenging to assemble an integrated genetic map. New technologies that can reveal conservative polymorphism at the inter-genus level are required to obtain fully integrative maps.

The genetic maps were updated by integrating DArTseq markers with two SNP platforms: DArTseq-derived SNPs and the Infinium 65k SNP array. Notably, the distribution of these markers was non-uniform across the genome, revealing a technology-dependent preferential clustering. SNPs from the Infinium array colocalized predominantly within regions dense with DArTseq markers, resulting in highly saturated genomic intervals. Conversely, DArTseq-derived SNPs failed to fully integrate with the DArTseq, clustering independently into distinct regions characterized by lower marker density. This technological fragmentation effectively partitioned several chromosomes into independent subgroups *a* and *b* (e.g., linkage groups CS.1a, CS.3a, CS.4a, CS.6b, CS.9b, PT.7a, and PT.9a, which were formed exclusively by DArTseq-derived SNPs).

This separation led to an increase of approximately 3000 cM for *C. sunki* and 2300 cM for *P. trifoliata*, metrics considerably higher than those typically reported in citrus literature (Curtolo et al., 2017; Huang et al., 2018). Although this map inflation reflects a methodological challenge in calculating recombination fractions across distinct platforms, retaining these loci was a strategic choice aimed at maximizing QTL mapping across the maximum number of loci. The biological validity and robustness of this assembly are supported by the high consistency in marker ordering observed across chromosomes 1 to 5 between the CS and PT maps (**Supplementary Figures 3**-**7**).

In this study, we utilized the chromosome-level, phased genome of *Citrus sinensis* cv. Valencia (assembly DVS_A genome v1.0) (Wu et al., 2023) to identify the positions of markers by chromosome. To our knowledge, this manually curated *C. sinensis* genome, which is available in the NCBI RefSeq database, provides the most accurate representation of citrus chromosomes currently available. However, *C. sinen*sis belongs to a third species, and the genome may not fully represent both parents. During the ordering procedure, we compared two sequences: one representing the *C. sinensis* physical chromosome and a second obtained by the ordering algorithm. Besides chromosome 8 for CS, all the others in our ordering provided a higher likelihood value than the physical one, indicating that rearrangements could have occurred during species differentiation.

### 4.2 Phenotypic data and QTL mapping:

Acquiring precise phenotypic data on plants’ response to HLB is quite challenging for several reasons: first, there is no single main response variable to define tolerance or susceptibility levels; second, there is no commercial resistant genotype to serve as a standard for evaluation. In this scenario, several authors have used Ct values obtained by qPCR to estimate *C*Las quantification and evaluate plant resistance (Huang et al., 2018; Soratto et al., 2020; Alves et al., 2021; Huang et al., 2023). Here, in this work, Ct was the trait used to evaluate the full-sib cross. However, environmental factors, bacterial development, and plant genotype may strongly influence this trait (Ramadugu et al., 2016; Chen et al., 2022a; Huang et al., 2023). To provide more substantial results, data from four different essays (two on greenhouse conditions and two on field conditions) over the years were used in this work. While differences between greenhouse and field conditions were not discernible, heritability has increased over the course of the essay period, suggesting a learning curve for evaluating HLB. This complex disease demands refined protocols to yield high-quality results.

The long juvenile period and the large experimental area are issues for citrus genetics studies. Regarding the CVg and the number of evaluated genotypes (**Table 2**), one notes that assays with different sample sizes provided similar results for the sampled genetic variability, including greenhouse (2021) and Field conditions (2014 and 2018). This result suggests that the HLB response can be verified even by evaluating a limited number of genotypes. Also, the genetic correlation between assays was estimated to be low, suggesting that interaction may have occurred due to several factors, such as climatic conditions, plant aging, and disease levels in the sampled plants. Huang et al. (2023) evaluated intergeneric crosses between trifoliate and sweet oranges’ responses to HLB over three years in field conditions, finding low heritability for *C*Las titer and low correlation with other phenotypic metrics assessed, reinforcing the sensitivity of this trait to the spatially and temporally variable distribution of the bacteria, which can also be influenced by environmental conditions.

QTL analysis also provided an updated view of phenotypic data: the heritability for Field-2014 was 72%, and all the mapped QTL captured 69% (CS map) and 31% (PT map); in this case, most phenotypic variation was captured by the association of QTL and markers. However, in Field-2018, heritability was 82%, but only 34% (CS map) and 17% (PT map) were explained by the QTLs.

The difference between these values needed to be interpreted with caution since the limited sample size in Field-2014 (66 genotypes) compared to Field-2018 (265 genotypes) can bias QTLs effects by inflating the captured genetic variance, known as the Beavis effect (Xu, 2003). This interpretation is corroborated by the fact that the CVg for both trials was similar (**Table 2**). For Greenhouse-2009, the QTL mapped in the CS map captured more variation (72%) (**Table 3**) than the phenotypic heritability (62%) (**Table 2**). In these three scenarios, the CS parent contributes more to the HLB variation than the PT parent. The only situation in which inversion occurred was in Greenhouse-2021, where QTL identified in the PT and CS maps accounted for 55% (**Table 4**) and 40% (**Table 3**), respectively. A sampling bias is observed in the Greenhouse-2021 trial, which presents a sample size and coefficient of variation (CVg) similar to those of Field-2014 (**Table 2**). However, in the Greenhouse-2009 trial, the sampling bias is more pronounced, since this experiment presented the lowest CVg among all those performed (**Table 2**).

Another interpretation about R2(%) was the comparison between CS and PT map. The QTL mapping model assumption is that for a given loci the parental is heterozygous (Qq), in which, one allele increases the phenotypic data and the other reduces (Gazaffi et al., 2014); probably CS-map covers more regions with heterozygous regions able to be detected under mapping procedure where a resistant genotype may be regarded by pyramiding these QTLs in a genotype.

Soratto et al. (2020) also evaluated the CS × PT population under greenhouse conditions and found 11 QTLs associated with *C*Las quantification. Three of these QTLs were identified in the CS map, accounting for 5.4 to 10.1% of the phenotypic variation (R2), and the other 11 QTLs were mapped in PT, with R2 ranging from 1.2 to 10.4%. However, a direct comparison between the QTLs mapped here and those reported by these authors is not possible due to differences in the physical positions and chromosome designations of the genetic maps. The QTL mapping presented by Soratto et al. (2020) used a previously published map (Curtolo et al., 2017), in which the pseudochromosomes of the draft genome of *C. sinensis* Valencia v1.0 (Xu et al., 2013) served as a reference. Due to low synteny between the reference genome used in this work and the previous references chosen by Curtolo et al. (2017) and Soratto et al. (2020), it is not possible to compare the QTLs identified, as neither the chromosome assignments nor the marker positions are consistent across the two genomes.

The main advantage of the QTL mapping approach applied here (Gazaffi et al., 2014) is the ability to estimate additive effects for both parents and dominance by exploiting the multipoint Hidden Markov Model, provided by the inclusion of 1:2:1 and 3:1 markers. When the genetic map contains regions with only partially informative markers, the conditional probabilities do not allow estimation of all genetic effects, and the model is adjusted to obtain the estimable ones, explaining why some effects were not inferred in **Tables 3**, **4**. In other words, of the 25 QTLs mapped for CS, in 12, the effect for the PT parent was estimable; and, the reciprocal also occurred, with nine effects for CS detected across the 19 QTLs identified in the PT map. Consequently, it was possible to identify QTL segregating in both parents, thereby improving understanding of the genetic control of HLB response and helping to understand the synteny between maps, i.e., the synteny between the CS.1b and PT.1a groups is verified by the location of the markers 100020178|F|0 and 100027786|F|0 (segregation 3:1) and by the identification of HLB response QTLs in both linkage groups.

The phenotypic data and QTL mapping indicated a pronounced genotype-by-environment (G×E) interaction, underscoring the complexities of dealing with *C*Las infection and, consequently, the progression of HLB disease. Based on phenotypic data, Ct values exhibited moderate heritability, reflecting a differential response to the disease across all trials. Conversely, low pairwise correlation coefficients were observed between individual trials, reinforcing the highly variable nature of the expression.

This G×E interaction is further evidenced by the QTLs. Out of 44 genomic regions associated with *C*Las, we identified four distinct genomic regions on chromosomes PT.1a (PT-HLB1 and PT-HLB10; **Table 4**), PT.3a (PT-HLB3 and PT-HLB17; **Table 4**), CS.4b (CS-HLB10 and CS-HLB15; **Table 3**), and CS.8 (CS-HLB6 and CS-HLB13; **Table 3**), where QTLs were colocalized across two different trials (**Supplementary Figures 3**, **5**, **6**, **10**), spaced by a maximum of 12.3 cM. Such minor fluctuations in QTL peak positions can be considered statistical artifacts arising from the use of partially overlapping subsets of genotypes, as sampling different cohorts from the same mapping population inherently limits resolution. Furthermore, the low number of stable QTLs shared between trials may suggest the impact of environmental conditions, such as temperature and water availability, on bacterial multiplication. Additionally, the mode of infection differs between environments; field trials involved natural transmission by psyllids, whereas greenhouse trials used artificial inoculation. This distinction fundamentally shapes bacterial colonization and the timeline of disease expression.

Considering that HLB is a complex pathosystem, this G×E indicates that different genomic regions are preferentially expressed under specific environmental and physiological conditions. Consequently, functional gene annotation became an essential tool to reveal convergent pathways across the detected QTLs, aligning with the primary goal of this study: to screen and identify candidate regions for future validation.

### 4.3 Functional annotation of candidate genes

Gene annotation involves the structural and functional characterization of genes and regulatory regions in a genome and can be classified into nucleotide-, protein-, and process-level annotation (Stein, 2001). The structural annotation of the reference *C. sinensis* genome (assembly DVS_A1.0, available at NCBI) enabled us to investigate the putative function of genes co-located with or in proximity to the identified HLB-responsive QTLs.

QTLs can represent the effect of genes and/or regulatory elements in proximal or distal regions of the genome, cis-regulation (Li et al., 2018) or trans-regulation (Lensink et al., 2025).

The functional annotation of genes and/or regulatory elements associated with QTLs depends on the correspondence between the genetic maps and the genome used as a reference; however, converting map distances expressed in centimorgans (cM) to physical distances expressed in base pairs (commonly kilobase pairs, kb) is challenging because there is no direct correlation between them (Temesgen and Yesuf, 2021). The recombination rate varies throughout a species’ genome (Brazier and Glémin, 2022; Casale et al., 2022; Payseur, 2025) due to heterochromatin distribution (Gaut et al., 2007) and several factors involved in meiosis (Wang and Copenhaver, 2018; Zou et al., 2024). Specific regions with higher recombination rates are called hotspots and have been reported in several species (Brazier and Glémin, 2024), making it difficult to accurately determine the cM/Mb ratio, especially in large and complex genomes such as those of higher plants (Gaut et al., 2007). In the scenario where the loci ordering in the CS and PT maps does not match the exact ordering of the loci in the *C. sinensis* genome, and considering the challenge of determining the cM/Mb ratio, and a QTL may contain thousands of base pairs in a genome, where dozens or even hundreds of genes may be present (Collins et al., 2008), a conservative approach was adopted to investigate candidate genes. This approach involved searching for gene sequences at the position in the *C. sinensis* genome corresponding to the molecular marker sequence colocalized with the QTL peak. When a gene was not identified at the exact position of the molecular marker, the search was extended to a maximum interval of 10 kb (5 kb upstream and 5 kb downstream). This process ensured that we identified the candidate genes most closely associated with the QTL peak regions.

Based on the parameters, 135 molecular markers were identified as significantly associated with the 44 QTLs, providing further opportunities for exploration in future studies. These candidate genes predominantly reflect biological functions related to responses to both biotic and abiotic stresses and to transposition (**Supplementary Tables 1**-**4**), as briefly presented below.

The identified genes indicate potential mechanisms by which citrus responds to HLB. Plants are particularly vulnerable to disruptions from environmental variations and biotic factors, which can influence their growth in both beneficial and detrimental ways. Hence, identifying stress factors and triggering signaling pathways are crucial for initiating defense responses and preserving plant homeostasis (Lamers et al., 2020). During the early phases of interactions between plants and pathogens, the host’s cellular systems need to detect the intrusion in order to initiate signaling pathways that lead to a defense response. Host proteins, such as cysteine-rich receptor-like kinases (CRKs), can recognize pathogen-associated molecular patterns (PAMPs) or effectors, along with signals of oxidative stress, which are decisive for establishing pattern-triggered immunity or effector-triggered immunity (Zeiner et al., 2023; Zhang et al., 2023). CRKs play a crucial role in regulating plant responses to both biotic and abiotic stress, as well as developmental stimuli (Zeiner et al., 2023; Zhang et al., 2023). We observed a notable link between the QTLs CS-HLB22 and PT-HLB12 and CRK10, suggesting stimulation of the plant’s response to bacterial invasion. There is still limited understanding regarding the recognition specificity of CRK10; nonetheless, an increase in the expression of this gene has been noted in sweet orange fruits infected with *C*Las, indicating that the pathogen’s attack, either directly or indirectly, led to alterations in its expression (Martinelli et al., 2015). The importance of CRK10 in plant resistance was demonstrated in rice, when inhibition of its expression compromised the NH1-mediated immunity (Chern et al., 2016). In grapes, suppression of CRK10 by the *Coniella diplodiella* effector CdE1 was also found to increase susceptibility to white rot (Liu et al., 2024b).

Another receptor that plays a role in defense mechanisms and is linked to the CS-HLB3 QTL is the leucine-rich receptor kinase MIK2 (MDIS1-interacting receptor-like kinase 2). This protein, which is overexpressed in *P. trifoliata* when attacked by *C*Las (Huang et al., 2023), serves as a regulator for responses to damage in the cell wall caused by the suppression of cellulose biosynthesis (Van Der Does et al., 2017). Additionally, MIK2 has been recognized as essential for the pattern-triggered immunity (PTI) seen in *Arabidopsis* against *Fusarium* spp (Coleman et al., 2021).

The activation of multiple defense mechanisms may be suggested by the TIR-NBS-LRR resistance proteins RPV1-like, DSC1, and N-like, which are associated with the QTL CS-HLB8 identified during field studies in 2018 (**Supplementary Tables 1**, **2**). RPV1 provides resistance against fungal (Shi et al., 2024) and viral (Gore et al., 2002) pathogens. The N protein, which is responsible for the hypersensitive response to TMV (Tobacco Mosaic Virus) (Whitham et al., 1994), has also been implicated in plant resistance to other viruses (Jan et al., 2000) and nematodes (Thies and Fery, 2000). DSC1 contributed to resistance against fungi (Li et al., 2019) and nematodes (Warmerdam et al., 2020). Although specific reports of these proteins in citrus’s defense against HLB are lacking, they may function in basal resistance. These proteins can initiate hypersensitive responses when specific pathogens attack the plant or support basal immunity to a range of pathogens, which can lead to compatible responses and disease progression, as observed by the N gene expression effect in the interaction between *Phaseolus vulgaris* and *Meloidogyne incognita* (Santini et al., 2016).

Under stressful circumstances, whether caused by living organisms or environmental factors, some alterations in the oxidative state of cells are observed, primarily due to an increase in the production of reactive oxygen species. Manifestation of this oxidative response includes peroxidases, which are associated with QTLs identified in the field in 2014 (CS-HLB1) and in the greenhouse trial in 2021 (PT-HLB16) (**Supplementary Tables 1**, **3**). These enzymes are responsible for detoxifying ROS and are also involved in plant development and cell wall strengthening, promoting plant defense against pathogen attack (Passardi et al., 2005). Peroxidase activity is relevant in the citrus response to HLB, with differential expression detected in several studies (Balan et al., 2018; Ramekar et al., 2025). Peroxidase accumulation was observed in the vascular tissue of plants of the susceptible variety Washington navel (*C. sinensis*) infected with *C*Las, which was evaluated in both the field and a greenhouse (Franco et al., 2020). Peroxidase overexpression was also observed in an HLB-tolerant hybrid generated by crossing the Marisol clementine with the Australian round lime (Ramekar et al., 2025). However, the members of the peroxidase family are induced, and their enzymatic activity varies according to the observed experiment (Balan et al., 2018; Franco et al., 2020; Ramekar et al., 2025).

Alterations in the cellular redox state are also suggested by the enzymes thioredoxin and thioredoxin reductase, linked to the QTLs PT-HLB15 and HLB19, as identified through greenhouse experiments (**Supplementary Table 3**).

These enzymes protect chloroplasts against oxidative stress caused by abiotic factors like water scarcity (Vanacker et al., 2018), high temperatures (Chae et al., 2013), and pathogen attacks (Mata-Pérez and Spoel, 2019). In citrus plants infected with HLB, the expression of thioredoxin was repressed, indicating the pathogen’s attempt to subvert the plant’s resistance mechanisms (Ribeiro et al., 2023; Zuo et al., 2023).

Another notable candidate gene regions were recognized as encoding reverse transcriptase domain-containing proteins, associated with CS-HLB1, CS-HLB3 and PT-HLB2, identified in 2014 (**Supplementary Tables 1**, **4**).

Plant proteins that contain the reverse transcriptase domain may be associated with telomerases (Sun et al., 2024), crucial in maintaining genomic stability, retrotransposons (Tang et al., 2005), or group II intron reverse transcriptase/maturases (Mohr, 2003), involved in genetic plasticity. Their expression level can be altered in the stress response. Telomerase reverse transcriptase plays a role in telomere preservation due to its protective effect against the accumulation of reactive oxygen species. Sun et al. (2024) found increased telomerase expression in *Medicago sativa* seeds subjected to aging and confirmed the role of telomerases in seed aging tolerance in genetically modified *A. thaliana*.

Other regions associated with transposition included retrovirus-related Pol polyproteins identified in the CS-HLB4 (**Supplementary Table 2**) and CS-HLB10 (**Supplementary Table 1**), which were detected under field conditions. These sequences synthesize proteins necessary for the transposition of LTR retrotransposons (Havecker et al., 2004). While the role of retrotransposons in the reaction to *C*Las has not been explored, there are findings of their varied expression in citrus plants affected by HLB (Lally et al., 2021). Transposable elements that function as cis-regulators for the expression of stress-responsive genes have been confirmed in *Arabidopsis thaliana* and *Solanum lycopersicum* (Deneweth et al., 2022). Additionally, cold-inducible retrotransposons play a role in regulating the expression of the Ruby gene in Sicilian blood oranges (*C. sinensis*) (Butelli et al., 2012).

## Data Availability

The datasets presented in this study can be found in online repositories. The names of the repository/repositories and accession number(s) can be found below: https://github.com/lucianesantini/, Citrus_raw_data.
