# Supplementary material for: Genetic mapping reveals QTLs associated with HLB response determined by Candidatus Liberibacter asiaticus quantification in a Citrus sunki × Poncirus trifoliata segregating population
Source: Front Plant Sci. 2026 Aug 17;17:1864793. doi: 10.3389/fpls.2026.1864793 (PMC13526623; doi:10.3389/fpls.2026.1864793)
Supplement: Supplementary file 1 [file Supplementaryfile1.zip › Supplementary_Figures.docx]

**Genetic mapping reveals QTLs associated with HLB response determined by *Candidatus* Liberibacter asiaticus quantification in a *Citrus sunki* × *Poncirus trifoliata* segregating population**

Luciane Santini-Gazaffi¹, Rodrigo Gazaffi^2^, Sinara Oliveira de Aquino¹, Dário Grattapaglia³, Marinês Bastianel¹, Mariângela Cristofani-Yaly¹*, Marcos Antonio Machado¹

^1^Instituto Agronômico de Campinas, Centro APTA Citros Sylvio Moreira, Cordeirópolis, SP, Brazil

^2^Universidade Federal de São Carlos, Centro de Ciências Agrárias, Departamento de Biotecnologia e Produção Vegetal e Animal, Araras, SP, Brazil

^3^ Empresa Brasileira de Pesquisa Agropecuária (EMBRAPA) - Recursos Genéticos e Biotecnologia, Brasília, DF, Brazil

*** Correspondence:**
Mariângela Cristofani-Yaly
mariangela@ccsm.br

Keywords: Greening, high-throughput markers, SNPs, QTL mapping, *Candidatus* Liberibacter asiaticus (*C*LAs)

Supplementary Figures

**Supplementary Figure 1**. Density map of *C. sunki* linkage groups (CS.1a to CS.9b). Linkage group numbering follows the nine *Citrus sinensis* chromosomes, according to their correspondence based on markers alignment to the *C. sinensis* reference genome (version DVS_A1.0; available at NCBI), using BLAStn algorithm. Linkage groups with GAP greater than 30 cM were separated into subgroups a and b. The heatmap represents linkage group density, ranging from 0.8 cM/locus (in red) to 30 cM/locus (blue).

**Supplementary Figure 2.** Density map of *P. trifoliata* linkage groups (PT.1a to PT.9b). Linkage group numbering follows the nine *Citrus sinensis* chromosomes, according to their correspondence based on markers alignment to the *C. sinensis* reference genome (version DVS_A1.0; available at NCBI), using BLAStn algorithm. Linkage groups with GAP greater than 30 cM were separated into subgroups a and b. The heatmap represents linkage group density, ranging from 0.8 cM/locus (in red) to 30 cM/locus (blue).

**Supplementary Figure 3.** Graphical representation of linkage groups CS.1b and PT.1a, focusing on positioning QTLs related to citrus response to HLB. CS-HLB1: QTL identified from field evaluations in 2014. PT-HLB1 and PT-HLB10: QTLs identified from field evaluations in 2014 and in greenhouse conditions in 2009, respectively. In black: markers mapped in *C. sunki* and *P. trifoliata*, allowing correspondence between the CS and PT linkage groups. In yellow: markers co-located with the QTLs.

**Supplementary Figure 4** Graphical representation of linkage groups PT.2b, CS.2a, PT.2a, and CS.2b, focusing on positioning QTLs related to citrus response to HLB. In the *P. trifoliata* linkage groups, QTLs were identified in field evaluations in 2014 (PT-HLB2) and in the greenhouse in 2009 (PT-HLB11 and PT-HLB12) and 2021 (PT-HLB16). In the CS.2a linkage group, the QTLs CS-HLB22 and CS-HLB23) were mapped from the greenhouse evaluations in 2021. In black: markers mapped in *C. sunki* and *P. trifoliata*, allowing correspondence between the CS and PT linkage groups. In yellow: markers co-localized with the QTLs.

**Supplementary Figure 5.** Graphical representation of linkage groups CS.3a, PT3.a, CS.3b, and PT.3b, focusing on positioning QTLs related to citrus response to HLB. In the CS.3a linkage group, the QTL CS-HLB7 was identified in the field trial performed in 2018, and CS-HLB24 and CS-HLB25 were identified in greenhouse evaluations in 2021. In the CS.3b linkage group, CS-HLB14 was identified in a greenhouse trial in 2009, and CS-HLB2 and CS-HLB8 were mapped from field trials in 2014 and 2018, respectively. In the *P. trifoliata* linkage groups, QTLs were identified in field evaluations in 2014 (PT-HLB3) and in 2018 (PT-HLB6) and in the greenhouse in 2021 (PT-HLB17 and PT-HLB18). In black: markers mapped in *C. sunki* and *P. trifoliata*, allowing correspondence between the CS and PT linkage groups. In yellow: markers co-localized with the QTLs.

**Supplementary Figure 6.** Graphical representation of linkage groups PT.4b, CS.4a, PT.4a, and CS.4b, focusing on positioning QTLs related to citrus response to HLB. In the *P. trifoliata* linkage groups, QTLs were identified in field evaluations in 2014 (PT-HLB4) and 2018 (PT-HLB7) and in the greenhouse in 2009 (PT-HLB13). In *C. sunki* linkage groups, the QTLs were identified in field trials in 2014 (CS-HLB3) and 2018 (CS-HLB9 and CS-HLB10) and in the greenhouse in 2009 (CS-HLB15 and CS-HLB16). In black: markers mapped in *C. sunki* and *P. trifoliata*, allowing correspondence between the CS and PT linkage groups. In yellow: markers co-localized with the QTLs.

**Supplementary Figure 7.** Graphical representation of linkage groups CS.5a, PT.5a, CS.5b, and PT.5b, focusing on positioning QTLs related to citrus response to HLB. In the *C. sunki* linkage groups, the QTL CS-HLB11 was identified in the field trial performed in 2018, and CS-HLB17 and CS-HLB18 were identified in greenhouse evaluations in 2009. In the *P. trifoliata* linkage groups, QTLs PT-HLB14 and PT-HLB15 were identified in the greenhouse in 2009. In black: markers mapped in *C. sunki* and *P. trifoliata*, allowing correspondence between the CS and PT linkage groups. In yellow: markers co-localized with the QTLs.

**Supplementary Figure 8.** Graphical representation of linkage groups CS.6a and PT.6a, focusing on positioning QTLs related to citrus response to HLB. CS-HLB4 and CS-HLB12: QTLs identified from field evaluations in 2014 and 2018, respectively. PT-HLB8: QTL identified in field evaluations in 2018. In yellow: markers co-located with the QTLs.

**Supplementary Figure 9.** Graphical representation of linkage groups CS.7a, PT.7a, CS.7b, and PT.7b, focusing on positioning QTLs related to citrus response to HLB. In the *C. sunki* linkage groups, the QTL CS-HLB5 was identified in the field trial performed in 2014, and CS-HLB19 and CS-HLB20 were identified in greenhouse evaluations in 2009. In the *P. trifoliata* linkage groups, QTLs PT-HLB5 e PT-HLB15 were identified in field trials in 2014 and 2018, respectively. In yellow: markers co-localized with the QTLs.

**Supplementary Figure 10.** Graphical representation of the linkage group CS.8. CS-HLB6 and CS-HLB13: QTLs identified from field evaluations in 2014 and 2018, respectively. In yellow: markers co-located with the QTLs.

**Supplementary Figure 11.** Graphical representation of the linkage group PT.9b. PT-HLB19: QTL identified in greenhouse trial in 2021. In yellow: marker co-located with the QTL.
